# Temporal efficiency evaluation and small-worldness characterization in temporal networks

**DOI:** 10.1038/srep34291

**Published:** 2016-09-29

**Authors:** Zhongxiang Dai, Yu Chen, Junhua Li, Johnson Fam, Anastasios Bezerianos, Yu Sun

**Affiliations:** 1 Singapore Institute for Neurotechnology (SINAPSE), Centre for Life Sciences, National University of Singapore, Singapore; 2 School of Computer Engineering, Nanyang Technological University, Singapore; 3 Department of Psychological Medicine, Yong Loo Lin School of Medicine, National University of Singapore, Singapore

## Abstract

Numerous real-world systems can be modeled as networks. To date, most network studies have been conducted assuming stationary network characteristics. Many systems, however, undergo topological changes over time. Temporal networks, which incorporate time into conventional network models, are therefore more accurate representations of such dynamic systems. Here, we introduce a novel generalized analytical framework for temporal networks, which enables *1*) robust evaluation of the efficiency of temporal information exchange using two new network metrics and *2*) quantitative inspection of the temporal small-worldness. Specifically, we define new robust temporal network efficiency measures by incorporating the time dependency of temporal distance. We propose a temporal regular network model, and based on this plus the redefined temporal efficiency metrics and widely used temporal random network models, we introduce a quantitative approach for identifying temporal small-world architectures (featuring high temporal network efficiency both globally and locally). In addition, within this framework, we can uncover network-specific dynamic structures. Applications to brain networks, international trade networks, and social networks reveal prominent temporal small-world properties with distinct dynamic network structures. We believe that the framework can provide further insight into dynamic changes in the network topology of various real-world systems and significantly promote research on temporal networks.

We live in an age of networks. Network models have been employed in the study of many complex systems, including biological systems, communication networks and ecological systems^1^. In a network analysis framework, individual members of the studied system (such as the brain regions in a brain network or the countries in a trade network) are represented by nodes, and the interactions among them are modeled as edges. Various graph theoretical measurements have been developed for investigating network topologies and to characterizing the underlying processes^2^,^3^,^4^,^5^,^6^. An important property that many real-world systems possess is small-worldness, which refers to an optimal combination of global integration and local segregation for efficient information processing^2^. Notably, most network models to date have been static and thus have neglected the possible temporal variations in the network topology. However, many real-world systems undergo structural changes over time, and time is, in fact, a critical factor in these network models^7^,^8^,^9^. For example, brain functional connectivity networks have been repeatedly shown to be highly dynamic^10^,^11^,^12^,^13^. Static network models tend to oversimplify these systems and therefore provide limited insights into their underlying network dynamics. In recent years, temporal network models, which incorporate time into static network models, have attracted substantial attention and have yielded some of the first quantitative insights in the search for a better understanding of the dynamic changes in network topologies in many real-world systems^8^,^14^. Given the paucity of research in this area, only a limited number of attributes of temporal networks, including reachability^15^,^16^, modularity^17^,^18^,^19^, and centrality^20^,^21^, have been explored.

Among the characteristics of static networks, global integration and local segregation are closely related to the efficiency of information processing in such a network. A unified framework for efficiency assessment in static networks, which consists of *global efficiency* and *local efficiency*, has been developed and widely applied^3^,^22^. Networks with information flows that are both globally efficient (compared with regular networks) and locally efficient (compared with random networks) are considered to possess the optimal small-world architecture^3^. In this study, we hypothesize that the small-world properties of static networks can be extended to temporal networks, and that systems with temporal small-world architectures should exhibit a special combination of topological and temporal structures that can facilitate temporally efficient information processing at both the global and local scales. To date, no generalized and robust framework for quantifying the efficiency of information transfer and quantitatively identifying the presence of temporal small-world structures in temporal networks has been proposed. Moreover, investigating the prominent network structures that contribute to the efficiency of a temporal small-world network will provide a more in-depth understanding of dynamic systems.

In this study, we introduce the concepts of *overall temporal global efficiency* and *overall temporal local efficiency*, which are measures of the efficiency of information exchange over time in a temporal network at the global and local scales respectively. Next, we revisit the various temporal random network models that have been proposed and define the temporal regular network architecture; these models are then employed as references for the identification of temporal small-world architectures and the characterization of dynamic network structures. Finally, we demonstrate the effectiveness of the framework by applying it to three types of complex real-world systems, i.e., brain functional connectivity networks, international trade networks (ITN), and social networks (specifically, human proximity networks). In addition, we explore the correspondence between the static small-worldness of an aggregated static network and the temporal small-worldness of the corresponding underlying temporal network. Here, we adopt several assumptions regarding the temporal networks under study. First, the time dimension is assumed to be discrete, which enables each temporal network to be conceptualized as a sequence of static graphs in which the edges are instantaneous. As a result of this assumption, at each time step, two nodes are considered to be connected as long as a static network path exists between them. Second, the connections in the networks are assumed to be binary and undirected. Many real-world systems (such as brain networks and trading networks) can be suitably represented by this type of temporal network model^8^.

## Methods

### Temporal network

We begin by introducing the basic concepts related to temporal networks. A temporal network of the type under study can be represented by *G *= {*G*_*t*_}_*t* = 1, 2, …, *T*_, where *G*_*t*_ is a static graph of size *N* × *N* (where *N* is the number of nodes), which corresponds to the snapshot of the network at time *t*, and *T* is the lifetime (which is equal to the total number of time steps) of the network^16^,^23^. The set of nodes in *G*_*t*_ is constant in time, whereas the set of edges in *G*_*t*_ may vary with time. In this work, we refer to the connections in the snapshot static graphs at specific time steps as *contacts* and to the connections in the graph aggregated over the entire network lifetime as *edges*, i.e., an edge exists between a node pair if they are connected by at least one contact throughout the network lifetime. Mathematically, a temporal network can be formulated as a 3-dimensional matrix with dimensions of *N *× *N *× *T*. The network matrices consist only of ones and zeros, which indicate the presence or absence, respectively, of a contact at a particular position. A *time-respecting path* from node *i* to *j* is a sequence of contacts with non-decreasing time labels that starts at *i* and ends at *j*^24^. A *time-respecting path* is constructed over both the spatial and temporal dimensions, while its static counterpart (a *path* in a static graph) involves only spatial configurations. The *temporal distance* (analogous to the *path length* in a static graph) from node *i* to *j* at time *t*, *τ*_*i→j*_(*t*), is defined as the smallest number of time steps required to reach node *j* from node *i* starting at time *t*^25^. Therefore, a temporal distance can be any positive integer, with the smallest value being 1 (when *i* and *j* are connected through a static path at time *t*) and the largest value being infinity (when no time-respecting path exists from *i* to *j* at time *t*). Note that although the term “distance” is included in the definition of temporal distance, it is a measure in the time domain. Moreover, because of the characteristics of the time-respecting path, the temporal distance is influenced by both the topology of each of the snapshot static graphs and the temporal structure of the network.

**Figure 1:**
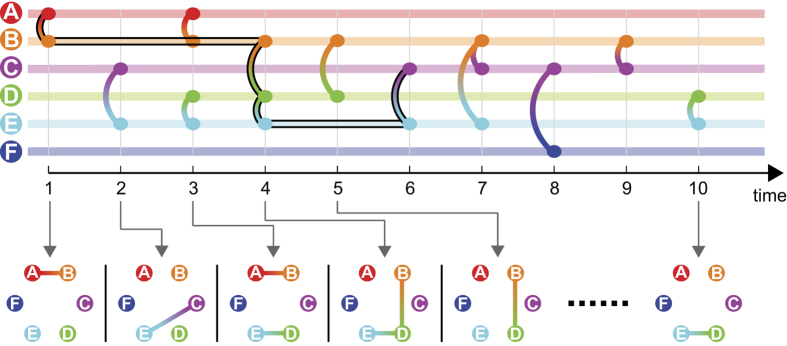
A sample temporal network (*N* = 6, *T* = 10) represented as a sequence of contacts, with selected snapshot static graphs shown at the bottom panel. A time-respecting path from node *A* to *C* at *t* = 1 is highlighted by the black strokes: {(*A*→*B*)_*t* = 1_, (*B*→*D*→*E*)_*t* = 4_, (*E*→*C*)_*t* = 6_}.

The basic concepts related to temporal networks are illustrated in a sample network with *N* = 6 and *T* = 10 in Fig. 1. In this sample network, a time-respecting path exists from node *A* to *C* at *t* = 1: {(*A*→*B*)_t = 1_, (*B*→*D*→*E*)_t = 4_, (*E*→*C*)_t = 6_} (highlighted by the black strokes in Fig. 1); because this path is the shortest time-respecting path from *A* to *C* at *t* = 1, the corresponding temporal distance is *τ*_*A→C*_(*t* = 1) = 6. The sample network in Fig. 1 demonstrates three characteristics of time-respecting paths and temporal distances in which they differ from their static-graph counterparts^25^: ***1*****) time dependency**, e.g., *τ*_*C→D*_(*t* = 1) = 3, whereas *τ*_*C→D*_(*t* = 3) = 8, and similarly, *τ*_*A→B*_(*t* = 1) = 1, whereas *τ*_*A→B*_(*t* = 4) = ∞; ***2*****) non-symmetry**, e.g., at *t* = 1, there is a time-respecting path from *A* to *C*, whereas no time-respecting path exists from *C* to *A* (*τ*_*A→C*_(*t* = 1) = 6, *τ*_*C→A*_(*t* = 1) = ∞), and similarly, *τ*_*C→D*_(*t* = 1) = 3, whereas *τ*_*D→C*_(*t* = 1) = 6; and ***3*****) non-transitivity**, e.g., at *t* = 1, time-respecting paths exist from *F* to *C* and from *C* to *D*, whereas no time-respecting path exists from *F* to *D*.

### Overall temporal efficiency measures

Efficiency measures for temporal networks, including the *temporal global efficiency* and the *temporal local efficiency*, were first proposed by Tang and colleagues^23^. These metrics are robust against disconnected node pairs; however, they are based only on the temporal distances measured at the beginning (*t* = 1) of the network lifetime (averaged over all node pairs) and thus neglect the time dependency of the temporal distances. Moreover, simply averaging the efficiency measures proposed in ref. 23 over the entire network lifetime will produce a biased outcome because time-respecting paths become increasingly unlikely to exist as time approaches the end of the network lifetime, and thus, those time-respecting paths that occurred at earlier time steps tend to inflate the results. To address this issue, Pan and Saramäki^25^ proposed a periodic boundary condition for calculating the average temporal distance: for each node pair, the first observed time-respecting path is appended to the end of the network lifespan. However, the formulation in ref. 25 assumes that no node pairs are disconnected, otherwise, the result would be infinity. In the remainder of this section, we introduce, for the first time, more generalized and unbiased efficiency measures for temporal networks.

Here, by extending the temporal distances considered in the efficiency calculation from the first time step into the entire network lifetime and adopting the periodic node-pair-specific boundary condition proposed in ref. 25, we calculate the ***overall temporal global efficiency*** (*E*^*t*^_*glob*_) by evaluating the inverse of the temporal distances over all node pairs and throughout the network lifetime:


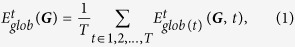


where *G* is the temporal network under consideration, *T* is the network lifetime, and *E*^*t*^_*glob*(*t*)_(*G*, *t*) represents the efficiency evaluated at time *t*:


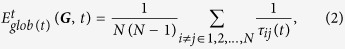


where *N* is the number of nodes in the network and *τ*_*ij*_(*t*) is the temporal distance from node *i* to *j* at time *t*. The values of both *E*^*t*^_*glob*(*t*)_ and *E*^*t*^_*glob*_ range from 0 to 1, and *E*^*t*^_*glob*_ = 1 when every node pair is connected through a static network path at every time step (every snapshot static graph is fully connected). Because 1/*τ* (instead of *τ*) appears in the calculation, this definition still holds in the presence of disconnected node pairs, in which case *τ* = ∞ for all *t* 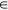 {1, 2, …, *T*} and such node pairs make no contribution to the overall network efficiency, *i*.*e*., 1/*τ* = 0. A larger value of *E*^*t*^_*glob*_ indicates a smaller average temporal distance among the nodes and fewer average time steps required for information from one node to reach another, corresponding to a more efficient overall information flow in the temporal network. Therefore, *E*^*t*^_*glob*_ is a strong indicator of how efficiently information is exchanged in a time-varying system.

Unlike in the definition of the temporal local efficiency in ref. 23, in which the neighbors of a node are obtained by aggregating all of its immediate neighbors (neighbors connected by a single contact) throughout the entire network lifetime, we define the neighbors of a node in a time-varying manner: neighbor identification for each node is performed at every time step. The notion of time-varying neighbors is important for accurately assessing the local information processing capability and fault tolerance of a temporal network because the set of neighbors that are directly affected by the removal of a node is time-dependent. In our formulation, the following two steps are repeated for each node at every time step: *1*) identifying the immediate neighbors of the node and forming the corresponding sub-temporal network (with dimensions of *n *× *n *× *T*, where *n* is the number of neighbors) and *2*) calculating the efficiency (*E*^*t*^_*glob*(*t*)_) of the sub-temporal network. Subsequently, the ***overall temporal local efficiency*** (*E*^*t*^_*loc*_) is derived by averaging the efficiency values over all nodes and all time steps:


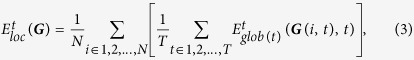


where *G*(*i*, *t*) is the sub-temporal network that consists of all immediate neighbors of node *i* at time *t* and preserves all contacts among these neighbors over the lifetime of the original network. *E*^*t*^_*glob*(*t*)_(*G*(*i*, *t*), *t*) represents the efficiency of *G*(*i*, *t*) at time t and is computed using equation (2). *E*^*t*^_*loc*_, whose values range from 0 to 1, measures the average efficiency of the temporal information exchange among the neighbors of a node and reflects the overall resilience of the temporal network to local failures caused by the removal of any node at any time point.

Notably, because the temporal efficiency measures proposed above are based on time-respecting paths and temporal distances, these two metrics depend on both the topological structure of the networks and the temporal configuration of the contacts in the networks, thereby quantifying the efficiency of information exchange over both the spatial and temporal dimensions.

### Temporal reference networks

In static network analysis, a small-world topology is discovered by comparing the efficiencies (global efficiency and local efficiency) of the network under study with those of random^26^ and regular lattice networks^22^,^27^. Analogously, to facilitate the quantitative assessment of temporal small-worldness based on the proposed temporal efficiency measures, reference temporal networks with highly random and highly regular architectures must be developed.

#### Temporal random networks

**Figure 2:**
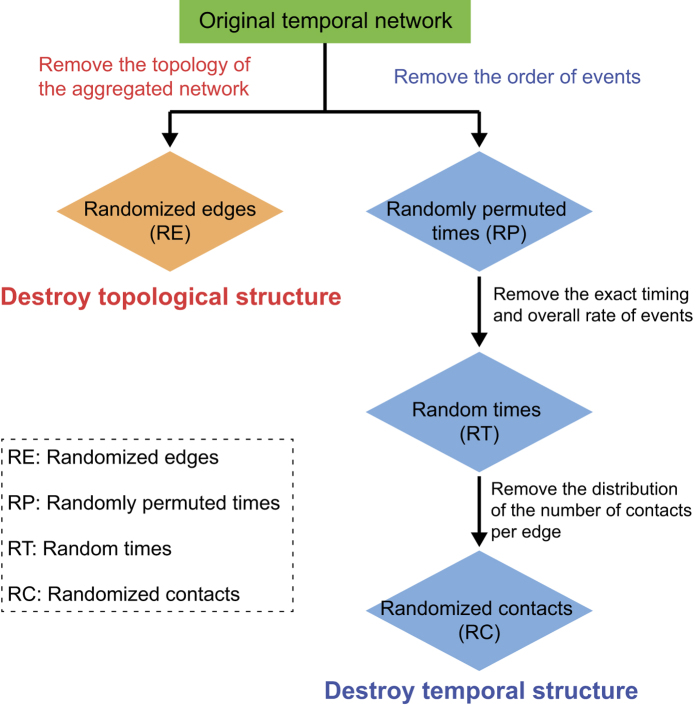
Summary of different randomization techniques adopted in this work, with emphasis on the particular types of structures removed from the specific networks. The combined usage of these algorithms constitutes a framework for evaluating the significance of different temporal network structures.

The richness of the complex structures in temporal networks allows the application of powerful temporal randomization techniques^7^,^8^,^14^, which can be used to produce reference networks for temporal small-worldness identification and to reveal various dynamic structures in temporal networks. The randomization techniques employed in this study include *randomized edges* (*RE*), in which all edges in the time-aggregated network are randomly rewired under certain constraints; *randomly permuted times* (*RP*), in which the timestamps of different edges are randomly swapped; *random times* (*RT*), in which all timestamps in each node pair are randomly redistributed; and *randomized contacts* (*RC*), in which all contacts in the network are randomly redistributed. As illustrated in Fig. 2, *RE* destroys the topological structures of the temporal network, whereas the other techniques progressively remove the temporal structures. The importance of different network structures in terms of their contributions to the network’s information processing efficiency can be evaluated by successively removing them by means of various temporal randomization techniques and comparing the temporal efficiency measures of the resultant random networks with those of the original network. The combination of *RE* and *RC* destroys most structures in a network; this combination is therefore employed to produce the reference random network for temporal small-worldness identification. More detailed descriptions (including pseudocode) of the algorithms for the different temporal randomization techniques are presented in the Supplementary Information.

#### Temporal regular network

**Figure 3:**
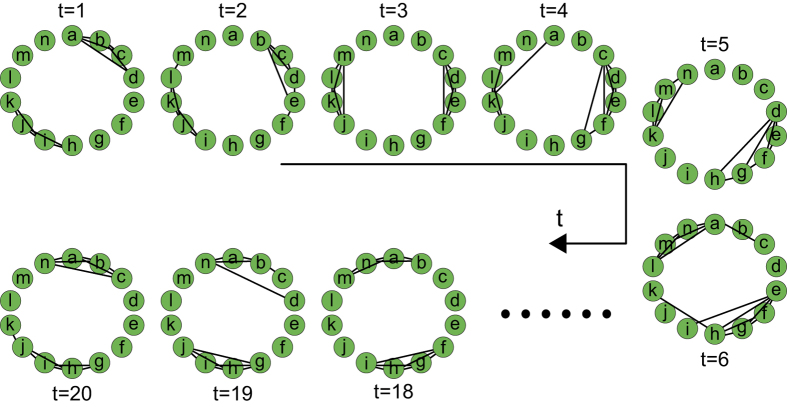
An example temporal regular network (*N* = 14, *T* = 20).

Generalizing from the characteristics of static regular lattice networks, we define a temporal regular network such that the temporal distances in the network are small between neighbouring nodes and large between distant nodes. Therefore, in the 3-dimensional matrix representation of a temporal regular network, all contacts are expected to cluster around the space diagonals (two topologically opposite space diagonals are selected such that the temporal proximity between adjacent nodes is balanced). The network generation algorithm consists of three steps: *1*) categorizing all contact positions into different layers, *2*) repeatedly sampling contacts from a layer probability distribution until the total number of contacts reaches that of the original temporal network, and *3*) randomly filling each layer with the corresponding number of contacts derived in step *2*). The layer to which a contact position belongs is calculated based on the proximity of the position to the two space diagonals, with smaller layer numbers indicating positions closer to the diagonals. Topologically, the layers of contacts are distributed following a specific pattern, e.g., the contacts in the first layer connect two adjacent nodes, whereas those in the first two layers join each pair of three neighbouring nodes. All contact positions are classified into *N* – 1 layers, and the distribution of the layers depends on the number of nodes (*N*) and time steps (*T*) of the network. The layer probability distribution is defined such that when the layer number is incremented by 1, the probability of a contact falling into that layer decreases by *p*. As a result, the inner layers (closer to the space diagonals) have higher probabilities of being filled and the contacts that minimizes the temporal distances between neighbouring nodes are favoured, thereby leading to a temporally regular structure of the network. The resulting temporal regular network consists of two topologically opposite clusters of contacts that are “traveling” in a specific circular direction through time (as is evident in Fig. 3). The free parameter in the model, *p*, controls the degree to which the contacts cluster around the diagonals. After running the network generation algorithm and calculating the network efficiencies with different *p* values, we selected a value of 1/4 for this parameter (see the Supplementary Information for further details on the selection of *p*). An example temporal regular network (*N* = 14, *T* = 20) is shown in Fig. 3. The pseudocode of the generation algorithm for temporal regular networks is included in the Supplementary Information.

#### Temporal small-worldness identification and dynamic network structure characterization

By combining the temporal efficiency measures and the temporal reference models defined above, we can quantitatively characterize the temporal small-world architecture, which features an optimal combination of temporal information communication on both the global and local scales: a temporal network is temporally small-world if the network possesses a larger overall temporal global efficiency than the corresponding temporal regular network and a greater overall temporal local efficiency than the corresponding temporal random network (with *RE* and *RC* randomizations), i.e., *E*^*t*^_*glob,regular*_ < *E*^*t*^_*glob*_ < *E*^*t*^_*glob,random*_, and *E*^*t*^_*loc,regular*_ > *E*^*t*^_*loc*_ > *E*^*t*^_*loc,random*_. In addition, the different temporal random networks destroy specific dynamic network structures, thereby enabling the investigation of the significance of individual network architectures in terms of their contributions to the temporal efficiency of information transfer.

### Experimental data

To validate the effectiveness and generalizability of the proposed framework, we applied the proposed techniques to three representative types of temporal networks, namely, brain functional connectivity networks, ITNs, and social networks.

#### Brain functional connectivity networks

Temporal brain functional connectivity networks were constructed from the resting-state functional magnetic resonance imaging (fMRI) data recorded in two sessions of a longitudinal study involving 17 participants, which investigated the effect of mindfulness training in older adults with mild cognitive impairment (MCI). Each network consisted of nodes representing 14 brain regions (which constituted the default mode network^28^) and 94 time steps representing the network lifetime. A range of sparsity values (referring to the preservation of the top *n* contacts in each static graph, *n* = 3, 4, …, 28) were applied to obtain binary temporal networks, and temporal random and regular networks were generated. For each of the temporal brain networks, the temporal efficiency measures were calculated and then integrated over the entire sparsity range to examine the presence of significant between-session differences.

#### International trade networks

Using the United Nations commodity trade database (UN COMTRADE^29^), we built a temporal ITN that consisted of 17 countries (the network nodes) and spanned 21 years (the network lifetime); the contacts were weighted based on the commodity-aggregated trade values and were assumed to be undirected. Subsequently, a series of sparsity values (representing the preservation of the top *n* contacts in each static graph, *n* = 10, 11, …, 30) were employed to produce different binary temporal networks.

#### Social networks

Temporal social networks were established using a dataset obtained from the SocioPatterns Project (http://www.sociopatterns.org/), which comprises the physical proximity data of 113 conference participants (corresponding to 113 nodes) over approximately 59 hours at the ACM Hypertext 2009 conference in Turin, Italy, from June 29 to July 1, 2009^30^. Further details about these data are provided in ref. 30, 31, 32, 33 and the Supplementary Information. Three binary aggregated temporal proximity networks were obtained by applying three different aggregation time windows (half-hour, one-hour and two-hour windows), after which the windows with no contacts were removed, resulting in networks with 72, 38, and 20 time steps, respectively. In addition to the temporal small-world characteristics of these networks, the impacts of temporal persistence (the degree of similarity between adjacent snapshot networks) and the choice of aggregation window on the temporal network efficiencies were also explored.

For each of the real-world temporal networks described above, the corresponding temporal random and regular networks were generated, and the temporal efficiency measures were calculated for both the original networks and the reference networks. More detailed descriptions of the network construction process for all three types of networks are presented in the Supplementary Information.

## Results

### Brain functional connectivity networks

**Figure 4:**
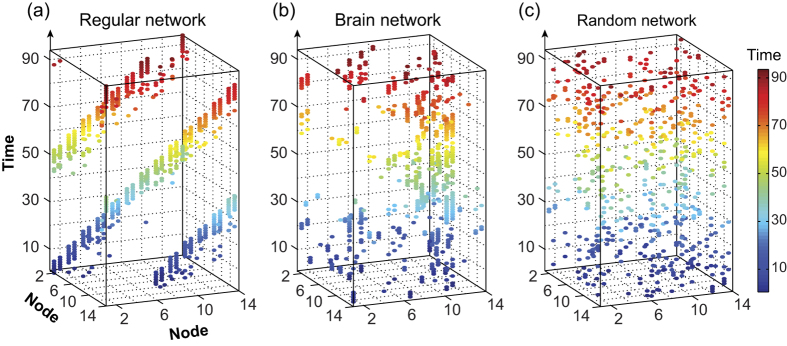
Spatio-temporal views of (**b**) one of the temporal brain networks (3 contacts preserved in each static graph), (**a**) the corresponding temporal regular network and (**c**) the corresponding temporal random network (with *RE* and *RC* randomizations).

**Figure 5:**
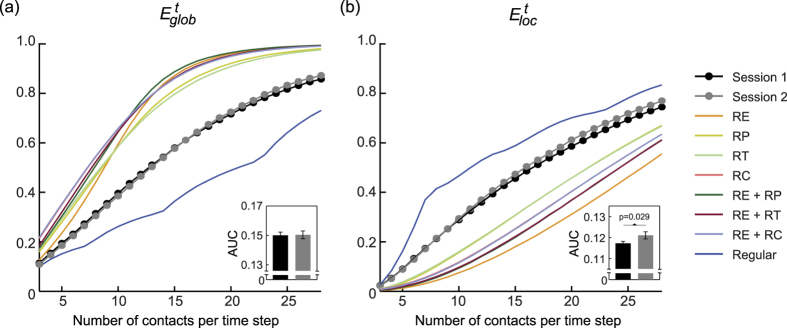
(**a**) Overall temporal global efficiency and (**b**) overall temporal local efficiency of the brain functional connectivity networks in both sessions (averaged over all subjects in each session), and those of the corresponding temporal regular and random networks (each efficiency value of the reference networks is averaged over all subjects, all iterations, and both sessions). Horizontal axis represents the different number of contacts preserved in each snapshot static graph. The integrated temporal efficiency measures (over the entire sparsity range) are shown at the bottom of the corresponding plot (mean ± standard error for each session), and the p value is displayed for the metric showing statistically significant session difference (*E*^*t*^_*loc*_).

Figure 4 shows the spatio-temporal structure of one of the temporal brain networks (with 3 contacts preserved in each static graph), along with the corresponding temporal regular network and one of the temporal random networks (with *RE* and *RC* randomizations; see Supplementary Fig. S2 for the other random networks). The *E*^*t*^_*glob*_ and *E*^*t*^_*loc*_ of the brain networks in both sessions and the corresponding temporal regular and random networks, averaged over all subjects, are plotted in Fig. 5. The efficiency measures integrated over all sparsity values (corresponding to the areas under the metric curves) are presented in Supplementary Table S1 to highlight the quantitative differences among the different types of networks. It can be observed from the figure that within the considered sparsity range, the temporal functional connectivity networks are both globally efficient (compared with the temporal regular network) and locally efficient (compared with the temporal random network with *RE* and *RC* randomizations); these findings are indicative of an optimal temporal small-world architecture. Moreover, as revealed in Fig. 5, all of the temporal randomization techniques resulted in significant variations of the temporal efficiencies of the brain networks, indicating the presence of diverse special network structures, the removal of any one of which severely affects the efficiency of temporal information communication on both the global and local scales. Furthermore, as shown in Fig. 5, a statistically significant increase was revealed in the integrated *E*^*t*^_*loc*_ (p = 0.029, one-sided permutation test) after the 3-month training, whereas no significant change was discovered in the integrated *E*^*t*^_*glob*_.

### International trade networks

**Figure 6:**
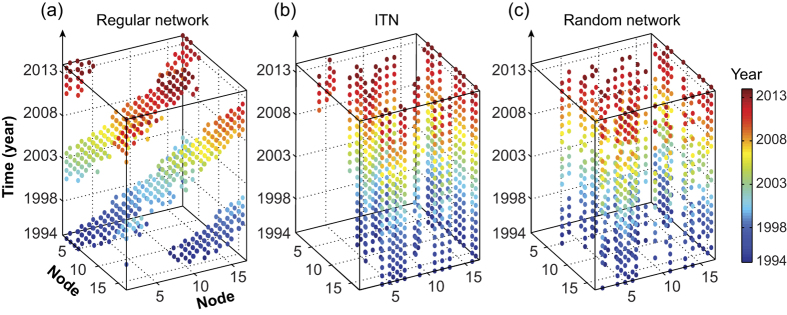
Spatio-temporal views of (**b**) one of the temporal international trade networks (17 contacts preserved in each static graph), (**a**) the corresponding temporal regular network, and (**b**) the corresponding temporal random network (with RE and RC randomizations). The nodes 1–17 correspond to the countries: Argentina, Australia, Brazil, Canada, China, France, Germany, Indonesia, Italy, India, Japan, Rep. of Korea, Mexico, Saudi Arabia, Turkey, the United Kingdom, and the United States.

**Figure 7:**
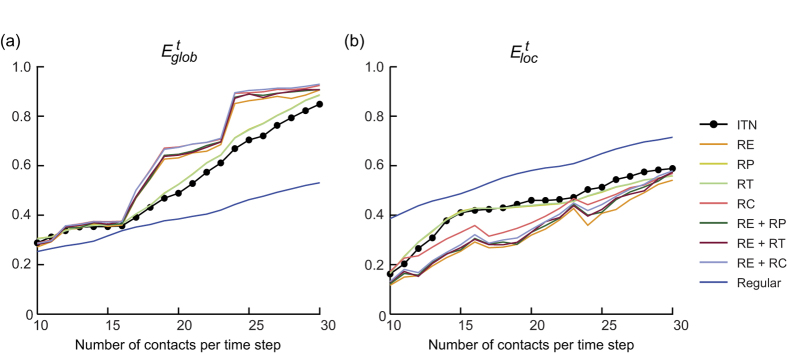
(**a**) Overall temporal global efficiency and (**b**) overall temporal local efficiency of the international trade networks, and those of the corresponding temporal regular network and the corresponding temporal random networks. Horizontal axis represents the different number of contacts preserved in each snapshot static graph.

Figure 6 presents a spatio-temporal view of one of the ITNs (with 17 contacts preserved in each snapshot static graph), together with the corresponding temporal regular network and one of the temporal random networks (with *RE* and *RC* randomizations). The *E*^*t*^_*glob*_ and *E*^*t*^_*loc*_ values of the temporal ITNs and all reference networks are plotted in Fig. 7. The efficiency values integrated over the entire sparsity range are presented in Supplementary Table S2 to illustrate the quantitative differences among the different types of networks. As shown in the figure, within the considered sparsity range, an optimal temporal small-world architecture was revealed in the temporal ITNs through the comparison of their temporal efficiencies with those of the temporal regular network and temporal random network (with *RE* and *RC* randomizations). Two major observations can be made by comparing the efficiency measures between the ITNs and the different corresponding temporal random networks (Fig. 7): *1*) in terms of the temporal structures, *RP* and *RT* caused insignificant changes to the efficiency measures, whereas *RC* significantly altered the temporal network efficiency, and *2*) in terms of the topological structures, *RE*, both alone and when combined with other techniques, resulted in significant variations in *E*^*t*^_*glob*_ and *E*^*t*^_*loc*_.

### Social networks

**Figure 8:**
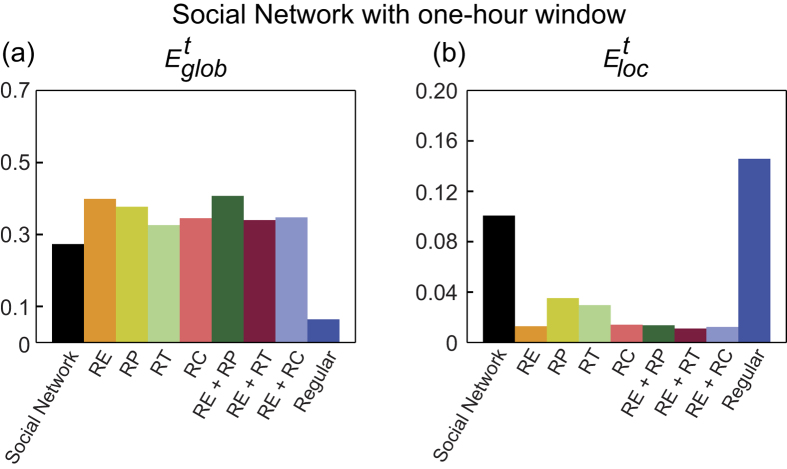
(**a**) Overall temporal global efficiency and (**b**) overall temporal local efficiency of the temporal proximity network constructed with one-hour aggregation window, and those of the corresponding temporal regular network and random networks.

The *E*^*t*^_*glob*_ and *E*^*t*^_*loc*_ values of the temporal human proximity network constructed with one-hour aggregation window, together with those of the corresponding reference networks, are shown in Fig. 8. The efficiency measures of the networks constructed with half-hour and two-hour windows are presented in Supplementary Fig. S3. As shown in these figures, for all three aggregation time windows, comparisons of the efficiency measures of the proximity networks with those of the temporal random and regular networks reveal a temporal small-world architecture in all three networks. Moreover, for all time windows, the *RP*-randomized networks exhibit reduced temporal persistence (Supplementary Table S3), together with increased *E*^*t*^_*glob*_ and decreased *E*^*t*^_*loc*_ (Fig. 8 and Supplementary Fig. S3). Furthermore, lengthening the aggregation window improves the temporal efficiency measures both globally and locally (Supplementary Table S4) through increasing the average nodal degree of the network (Supplementary Table S5). More detailed descriptions of the results of the human proximity network analysis are presented in the Supplementary Information.

## Discussion

Recent advances in temporal network analysis methods permit quantitative investigations of dynamic network structures and provide effective tools for understanding, predicting and optimizing the behaviour of complex systems that undergo topological variations over time. In the current work, we have presented a generalized and robust analytical framework for temporal network characterization and have demonstrated the reliability of that framework by applying it to three types of complex real-world systems: brain functional connectivity networks, ITNs, and social networks. In addition, we also investigated the correspondence between the temporal small-worldness of a temporal network and the static small-worldness of the corresponding aggregated static network and presented detailed analysis and discussions in the Supplementary Information.

### Brain functional connectivity networks

To date, most graph theoretical studies of the brain functional connectivity networks have been conducted under the assumption of stationary network characteristics^4^,^34^. However, the functional coupling among different brain regions has been discovered to be highly dynamic^10^,^11^,^12^,^13^. Therefore, the analytical framework introduced in the current study, which incorporates the temporal variations in functional connections into the graph theoretical framework, has the potential to provide deeper insight into the intrinsic organization of brain functional networks.

In previous studies, through the application of graph theory, small-world topologies have been discovered in brain functional connectivity networks, featuring both dense local clustering of neighbouring brain regions and short path lengths between distant brain regions^22^,^27^,^35^. In this work, to account for the dynamic variations in functional connections, we extended the definition of small-worldness to temporal brain networks and revealed the optimal temporal small-world organization of brain functional connectivity networks (Fig. 5). The results of this study therefore demonstrate that the overall dynamic exchange of information among brain regions is efficient and that the special temporal structure of the brain functional networks facilitates effective coordination of various brain regions for globally integrated brain functions; meanwhile, the transfer of information over time among neighbouring brain regions is also efficient, facilitating the effective temporal functional specialization of the temporal brain network.

Comparisons between the studied brain networks and the different corresponding temporal random networks revealed that all of the considered temporal randomization techniques could significantly alter the temporal network efficiency (Fig. 5). Therefore, the functional connectivity network is highly dynamic, and the results indicate that temporally efficient communication of information in the brain network is facilitated by the combination of various prominent network architectures, including the topology of the aggregated brain network, the order of the functional connectivity events, the number of preserved connections in each time window, and the distribution of the total number of contacts between each pair of brain regions. Each of these dynamic structures carries information on the fundamental organization of the temporal functional connectivity network, the disruption of which will cause the optimally efficient behaviour of the temporal functional connectivity network to degrade. This finding further corroborates previous studies discovering the abundant dynamics contained in brain functional connectivity networks^10^,^11^,^12^,^13^.

More interestingly, as revealed in Fig. 5, the integrated *E*^*t*^_*loc*_ was significantly improved after 3 months of mindfulness training. This finding might suggest that mindfulness practice significantly improves the efficiency of local temporal information processing and the fault tolerance of the temporal functional connectivity network. Therefore, our finding provides further neuroimaging evidence to support the notion that mindfulness practice could improve the cognitive function in older adults with MCI^36^. More importantly, this result also demonstrates the effectiveness of the temporal efficiency metrics introduced in this work in revealing the underlying dynamic characteristics of brain functional networks.

### International trade networks

Recent advances in complex network theory have also sparked new interest in ITNs^37^,^38^,^39^. Temporal information is an inherent component of international trade relationships, because the spreading of events (such as economic crises) over time in an ITN can have dramatic worldwide impacts^40^,^41^. Therefore, temporal network analysis, which integrates time into the conventional network models, has the potential to uncover more in-depth characteristics of international trade.

Using the analytical framework introduced in this study, a temporal small-world architecture was discovered in the investigated ITNs (Fig. 7), which suggests that the trades among these 17 countries are temporally efficient at the global network scale, featuring short average temporal path lengths between all pairs of countries, and that the trade network is also efficient at the local scale and resistant to local network failures such as the removal of certain countries from the network. This finding might provide further evidence for the coexistence of globalization and regionalization in international trade^42^,^43^.

Moreover, by examining the variations in the temporal network efficiencies induced by different temporal randomization techniques, two prominent dynamic network structures were revealed. First, the temporal network efficiencies were found to be minimally affected by the *RP* and *RT* techniques; however, they are significantly altered by the *RC* technique (Fig. 7). This phenomenon reflects the significance of one of the network structures, namely, the distribution of the number of contact events between each node pair, which is preserved by *RP* and *RT* but destroyed by *RC*. The prominence of this network structure results from strong bias in the contact distribution and the existence of certain specific connections that appear highly frequently over time, which are evident in Fig. 6 and directly reflect the large, steady trade volumes between certain trade partners. For example, as shown in Fig. 6, when the top 17 trade partners in each year are retained, 12 trading pairs are consistently present in the network every year, such as the USA and China as well as the USA and Canada. This finding also serves as novel quantitative evidence for the strong persistence over time of the topology of ITN^44^,^45^, which results from the bias of the trades towards some specific pairs of countries. Secondly, the substantial alterations in the temporal efficiencies caused by *RE* randomization suggest that the aggregated structure of the temporal ITN significantly contributes to the efficiency of international trade. This phenomenon demonstrates that the trade relationships among these countries are formed such that the overall ITN performance is optimized. Another noteworthy pattern in Fig. 7 is the discontinuous jumps in the curves of some of the random networks (*RE*, *RC*, *RE* + *RP*, *RE* + *RT* and *RE* + *RC*). This phenomenon can be explained as discontinuities in the rate of increase of the number of edges in the aggregated graph as the number of contacts increases in each time step (this behaviour can be further attributed to the persistence of contacts) and the relative sensitivity of the *RE* and *RC* algorithms to the aggregated number of connections.

### Social networks

In the analysis of physical proximity networks, although static network analysis has provided deep insight into the characteristics of these networks, the dynamic network properties can only be assessed by integrating the temporal variations in human contacts into these network models^46^,^47^. Technological advances in recent years have significantly expanded the availability of high-resolution human proximity data, thereby greatly facilitating the temporal network analysis of proximity networks^48^.

The observed presence of temporal small-world architecture in the proximity networks investigated in this study might indicate that the temporal evolution and topological arrangement of the interpersonal interactions is optimized such that both the overall interactions among all conference participants and the localized communications within small communities are organized to be efficient. This discovery complements the observation of static small-worldness in the aggregated network of the same dataset^30^, suggesting that both the topological and temporal structures of the proximity network are optimally arranged for efficient information exchange. In addition, the temporally persistent structure in these social networks, which is quantified by adjacency correlation^47^, causes the efficiency of interpersonal communication to be deflated globally yet inflated locally. In networks with relatively high temporal persistence (high degree of similarity between adjacent snapshot static graphs), the temporal global efficiency is reduced because the strong similarity between adjacent snapshot networks increases the number of required time steps needed to build a time-respecting path between two distant nodes; the temporal local efficiency is enlarged due to the temporal stability of densely connected local communities. Moreover, lengthening aggregation window in the temporal proximity network analysis was discovered to boost the temporal network efficiency both globally and locally, which might be explained by the increasing connection density at each time step. More detailed discussions of the results of the social network analysis are provided in the Supplementary Information.

### Effectiveness and generalizability of the proposed analytical framework for temporal networks

The analytical framework for temporal networks proposed in this study is effective and generalizable for three reasons. First, the proposed temporal efficiency measures can be used to measure the efficiency of temporal information exchange among the network nodes and to reveal the underlying network characteristics. For example, in the brain network analysis, *E*^*t*^_*loc*_ was discovered to be a neurobiologically meaningful metric due to its ability to quantify the structural alterations of the studied brain functional networks that resulted from mindfulness practice. Secondly, the framework permits the quantitative identification of temporal small-world architectures in complex systems that undergo topological variations over time. Compared with static small-world network analysis, the ability to identify the presence of temporal small-worldness in real-world systems has the potential to promote a more comprehensive and in-depth understanding of the overall information transfer occurring in the underlying dynamic processes of these systems. Third, the contributions of different dynamic network structures to the temporally efficient performance of a temporal network can be quantitatively evaluated. Many real-world systems are rich in dynamics, and the overall efficient behaviour of these systems may be the result of the interactions among many different prominent network structures. For example, in the ITN analysis, both the distribution of the number of significant trade events between each pair of trade partners and the time-aggregated structure of the temporal trade network were discovered to be special configurations of the investigated temporal ITN, which combine to facilitate optimally efficient international trades among the countries involved. We believe that taken together, these results suggest that the analytical framework proposed in this study is a beneficial means of temporal network characterization that is applicable in studies of diverse types of real-world systems that can be appropriately modelled as temporal networks.

### Methodological considerations

In this work, we assumed the contacts in the investigated temporal networks to be unweighted and undirected. However, weighted and/or directed network models have also been widely applied in the study of many real-world systems using static graph theoretical methods. For example, the edges in brain functional connectivity networks can be weighted by the magnitudes of the corresponding inter-regional correlations, and directionality can be added by examining the causal associations between pairs of brain regions^4^,^49^; in ITNs, the edges can be weighted by trade volumes^50^ and directed in accordance with import/export relationships^37^,^50^; and human proximity networks are often weighted by the total durations of the contacts^30^,^51^. Therefore, in future studies of temporal networks, new analytical tools can be developed by extending the method to consider weighted and/or directed contacts and modifying the definitions of the temporal efficiency measures accordingly.

When modelling a dynamic system as a series of snapshot static networks, an appropriate aggregation time window must often be selected, which may exert an important influence on the resulting network characteristics^8^,^47^,^52^. In the study of human proximity networks, we explored the properties of networks constructed with three different aggregation time windows, and both consistencies and differences were discovered. Although the main focus of the current study is the analysis, rather than the modelling, of temporal networks, future studies exploring the influence of the time windows used in the modelling of different systems might reveal more of the underlying dynamic characteristics of these systems.

## Conclusion

In this study, we presented a comprehensive analytical framework for temporal networks, which encompasses a robust assessment of the efficiency of temporal information exchange, the characterization of temporal small-worldness and the quantitative investigation of dynamic network structures. Subsequently, we applied the proposed analytical techniques to brain functional connectivity network, ITNs, and social networks, all of which exhibited temporal small-world architectures and distinct dynamic network structures, thereby demonstrating the effectiveness and generalizability of the proposed framework.

## Additional Information

**How to cite this article**: Dai, Z. *et al*. Temporal efficiency evaluation and small-worldness characterization in temporal networks. *Sci. Rep*. **6**, 34291; doi: 10.1038/srep34291 (2016).

## Supplementary Material

Supplementary Information
