# Supplementary material for: Temporal efficiency evaluation and small-worldness characterization in temporal networks
Source: Sci Rep. 2016 Sep 29;6:34291. doi: 10.1038/srep34291 (PMC5041081; doi:10.1038/srep34291)
Supplement: Supplementary Information [file srep34291-s1.pdf]

Supplementary Information: Temporal efficiency and small-worldness characterization in temporal networks

Zhongxiang Dai [1], Yu Chen [2], Junhua Li [1], Johnson Fam [3], Anastasios Bezerianos [1], Yu SUN [1*]

[1] Singapore Institute for Neurotechnology (SINAPSE), Centre for Life Science, National University of Singapore, Singapore

[2] School of Computer Engineering, Nanyang Technological University, Singapore

[3] Department of Psychological Medicine, Yong Loo Lin School of Medicine, National University of Singapore, Singapore

Contents

I. Temporal random networks
  1) Randomized edges (*RE*)
  2) Randomly permuted times (*RP*)
  3) Random times (*RT*)
  4) Randomized contacts (*RC*)
  5) Summary
II. Temporal regular networks
  1) Selection of the free parameter, *p*
  2) Network generation algorithm
III. Spatio-temporal view of all types of temporal networks (brain functional connectivity network)
IV. Construction of temporal brain functional connectivity networks
V. Construction of temporal international trade networks
VI. Construction of temporal social networks
VII. Integrated temporal efficiency measures
VIII. Results and discussion of temporal social networks
IX. Static *vs.* temporal small-world networks

I. Algorithms of different temporal random networks

We have implemented and assessed seven temporal randomization models (four randomization algorithms + three combinations of algorithms) in this work[1-3]. The randomization approaches can be generally categorized into two categories: (1) randomized edges (*RE*) destroys the topological structure of the temporal network; (2) the rest of the algorithms: randomly permuted times (*RP*), random times (*RT*) and randomized contacts (*RC*) remove different components of the temporal structure of the network. Specifically, in the aggregated graph (an ($N \times N$) matrix, with N representing the number of nodes and each entry of the matrix containing all the timestamps when the corresponding edge exists), *RE* modifies the graph topology, while *RP*, *RT* and *RC* only reorganizes the timestamps of the existing edges, leaving the aggregated graph topology unaltered. Temporal networks that are both topologically and temporally randomized can be obtained through the combination of *RE* with the other three techniques. More detailed descriptions on each of the temporal randomization techniques, including the pseudocode for network generation algorithms, are detailed in the following sections.

1) Randomized edges (*RE*)

RE is an extension of the common randomization technique for static graph which involves random edge rewiring while preserving the degree distribution and the connectedness of the network[4]. Equivalently, *RE* can be interpreted as first obtaining the aggregated graph, and then performing the same algorithm as that in static network on the aggregated graph with the difference that each edge carries the timestamps when being rewired[1]. In our formulation, an additional step is performed before the random rewiring: the edges are randomly swapped, because unlike binary static network, the edges in the aggregated graph carry additional information because of the possibly different timestamps. After *RE* is performed on a temporal network, the distribution of the contact sequences (the specific sequences of timestamps), the total number of contacts, and the connectedness of the aggregated network are preserved. Due to the random rearrangements of edges, in the aggregated network, disconnected (not linked by one connection) node pairs in the original network can become linked and previously directly connected may end up separated. Performing *RE* on a temporal network can be used to investigate the significance of the topological structure in the network as opposed to temporal structure.

The pseudocode of *RE* is shown below.

Algorithm: Randomized edges (*RE*)

Inputs: Temporal network ***G*** ($N \times N \times T$), each network entry represents the presence (1) or absence (0) of a contact

Outputs: Temporal random network, $\boldsymbol{G_{rand}}$ ($N \times N \times T$)

Begin

```
Derive a new network representation: G_t (N × N), where each entry contains all the timestamps of the node pair;
Define the total number of iterations: ITER (50 by default);
Find the set of node pairs, K, where at least one contact exists, i.e., K = {(i, j): i, j ∈ 1:N, length(G_t[i, j]) ≥ 1};
% Step 1: swap the timestamps of two randomly selected node pairs in K;
For i = 1 to ITER
    Randomly select two non-overlapping node pairs from K: (i1, j1), (i2, j2);
    Swap the timestamps of the two node pairs: swap G_t[i1, j1] with G_t[i2, j2];
Define the maximum number of attempts in each iteration: att_max (50 by default);
% Step 2: Randomly rewire the edges between the node pairs in K
For i = 1 to ITER
    Initialize the counter of the number of attempts: att = 0;
    While att < att_max
        Initialize a flag indicating whether rewiring should be performed: rewire = 1;
        Randomly select two non-overlapping and non-intersecting node pairs from K: (a, b), (c, d);
        Swap c and d with probability of 50%;
        If ((a, d) ∉ K) && ((b, c) ∉ K)
            If ((a, c) ∉ K) && ((b, d) ∉ K) % Test whether connectedness needs to be checked
                If rewiring will destroy the connectedness of the network
                    rewire = 0;
            If rewire == 1
                Rewire the timestamps of (a, b) to (a, d), i.e., move G_t[a, b] to G_t[a, d];
                Rewire the timestamps of (c, d) to (b, c) i.e., move G_t[c, d] to G_t[b, c];
                Exit the while loop;
        att = att + 1;
Convert G_t to the original network representation: G_rand (N × N × T);
```

Return G_{rand};

2) Randomly permuted times (*RP*)

In *RP* approach, the temporal structure of the original network is disrupted. In each iteration, two contacts at different node pairs are randomly selected; if the timestamps of the contacts are different and each timestamp is not present in the other node pair, the two contacts are swapped. After *RP* randomization, the order of the contact events are destroyed, while the number of contacts in each node pair, the number of contacts at each time step and the set of connected edges in the aggregated network are retained. Therefore, *RP* technique can be used to evaluate the impact of the order of events in the network.

The pseudocode of *RP* is shown below.

Algorithm: Randomly permuted times (*RP*)

Inputs: Temporal network ***G*** ($N \times N \times T$), each network entry represents the presence (1) or absence (0) of a contact

Outputs: Temporal random network, $\boldsymbol{G_{rand}}$ ($N \times N \times T$)

Begin

```
Derive a new network representation: G_t (N × N), where each entry contains all the timestamps of the node pair;
Define the total number of iterations: ITER (50 by default);
Find the set of node pairs, K, where at least one contact exists, i.e., K = {(i, j): i, j ∈ 1:N, length(G_t[i, j]) ≥ 1};
Calculate the total number of contacts in the network, C_sum;
Define the maximum number of attempts in each iteration: att_max (50 by default);
For i = 1 to ITER
    Initialize the counter of the number of attempts: att = 0;
    While att < att_max
        Randomly select two different node pairs from K: (i1, j1), (i2, j2);
        Randomly select a timestamp in (i1, j1): t1;
        Randomly select a timestamp in (i2, j2): t2;
        If (t1 ≠ t2) && (t1 ∉ G_t[i2, j2]) && (t2 ∉ G_t[i1, j1])
            Swap t1 and t2: add t1 to G_t[i2, j2] and t2 to G_t[i1, j1], remove t1 from G_t[i1, j1] and t2 from G_t[i2, j2];
            Exit the while loop;
        att = att + 1;
Convert G_t to the original network representation: G_rand (N × N × T);
Return G_rand;
```

3) Random times (*RT*)

In *RT* algorithm, the timestamps of the contacts in each node pair are randomly redistributed. As a result, the exact timing of the contacts and the overall rate of events (the number of contacts at each time step) are destroyed. The conserved network properties include the total number of contacts between each node pair and the set of connected node pairs in the aggregated network. *RT* can be used to investigate the effect of the overall rate of events in the temporal network, because compared with *RP*, *RT* introduces more randomness into the network by removing this network structure.

The pseudocode of *RT* is shown below.

Algorithm: Random times (*RT*)

Inputs: Temporal network $\boldsymbol{G}$ ($N \times N \times T$), each network entry represents the presence (1) or absence (0) of a contact

Outputs: Temporal random network, $\boldsymbol{G_{rand}}$ ($N \times N \times T$)

Begin

Derive a new network representation: G_t ($N \times N$), where each entry contains all the timestamps of the node pair;

Find the set of node pairs, K, where at least one contact exists, *i.e.*, $K = \{(i, j): i, j \in 1{:}N, \text{length}(G_t[i, j]) \geq 1\}$;

For each node pair $(i, j) \in K$

- Find the number of contacts: $c = \text{length}(G_t[i, j])$;
- Generate a randomly permuted integer sequence from 1 to T: r;
- Extract the first c elements of r: $s = r[1{:}c]$;
- Update G_t: $G_t[i, j] = s$;

Convert G_t to the original network representation: G_{rand} ($N \times N \times T$);

Return G_{rand};

4) Randomized contacts (*RC*)

RC randomization makes the network more temporally random than both *RP* and *RT*. *RC* is implemented by counting the total number of contacts in the network and redistributing equal number of contacts randomly among all connected node pairs (in the aggregated graph) and all time steps. After *RC* randomization, only the overall number of contacts in the network and the topology of the aggregated network are preserved. Compared with *RT*, *RC* further destroys the distribution of the number of contacts in each node pair, and thus can be employed in studying the role of this property in the network.

The pseudocode of *RC* is shown below.

Algorithm: Random contacts (*RC*)

Inputs: Temporal network $\boldsymbol{G}$ ($N \times N \times T$), each network entry represents the presence (1) or absence (0) of a contact

Outputs: Temporal random network, $\boldsymbol{G_{rand}}$ ($N \times N \times T$)

Begin

```
Find the set of node pairs, K, where at least one contact exists, i.e., K = {(i, j): i, j ∈ 1:N, length(G_t[i, j]) ≥ 1};
Calculate the total number of contacts in the network, C_sum;
Initialize an empty matrix to store the new network, G_rand (N × N × T);
% The following "for" loop ensures that the set of connected node pairs are unchanged
For each node pair (i, j) ∈ K
    Select a random time step: t ∈ 1:T;
    Assign a contact to the position: G_rand[i, j, t] = 1;
% The following "for" loop fills the remaining contacts (if any)
If C_sum > length(K)
    For i = 1 to (C_sum – length(K))
        Initialize a flag indicating whether a contact has been inserted: filled = 0;
        While filled == 0
            Select a random node pair: (i, j) ∈ K;
            Select a random time step: t ∈ 1:T;
            If the selected time step is not occupied, i.e. if G_rand[i, j, t] == 0
                Set the flag: filled = 1;
                Assign a contact to the position: G_rand[i, j, t] = 1;
Return G_rand;
```

II. Algorithms of temporal regular networks

1) Selection of the free parameter, *p*

In order to facilitate the desired behavior of the contacts (tending to fill the layers with smaller layer numbers), a probability model over the layers is developed such that the probability for a contact to fall into a layer decreases by *p* compared with the previous layer (with smaller layer number). Therefore, the probability mass function is a geometric series and the probability for a contact to exist in layer *l* is

$$p(l) = \frac{p^{l-1}}{\frac{p^{N-1}-1}{p-1}} \tag{S1}$$

As a result, inner layers (with smaller layer numbers) have larger probability of being filled, and the network favors the connections that minimize the temporal distances between neighboring nodes. During the network generation process, contacts are repeatedly sampled from the layer probability model and filled into the corresponding layers. The free parameter in the layer probability model, *p*, controls the contacts' degree of clustering around the diagonals, and therefore should be appropriately selected. For all three types of real-world networks analyzed in this study (brain networks, international trade networks and social networks), we employed different values of *p* in the temporal regular network generation model and the behaviors of the temporal network efficiencies are shown in Fig. S1. For all three types of networks, it can be observed from the figure that as the value of *p* decreases, minimal variations of the network efficiencies occur after the value reaches 1 / 4. Therefore, *p* = 1 / 4 is adopted in the case studies.

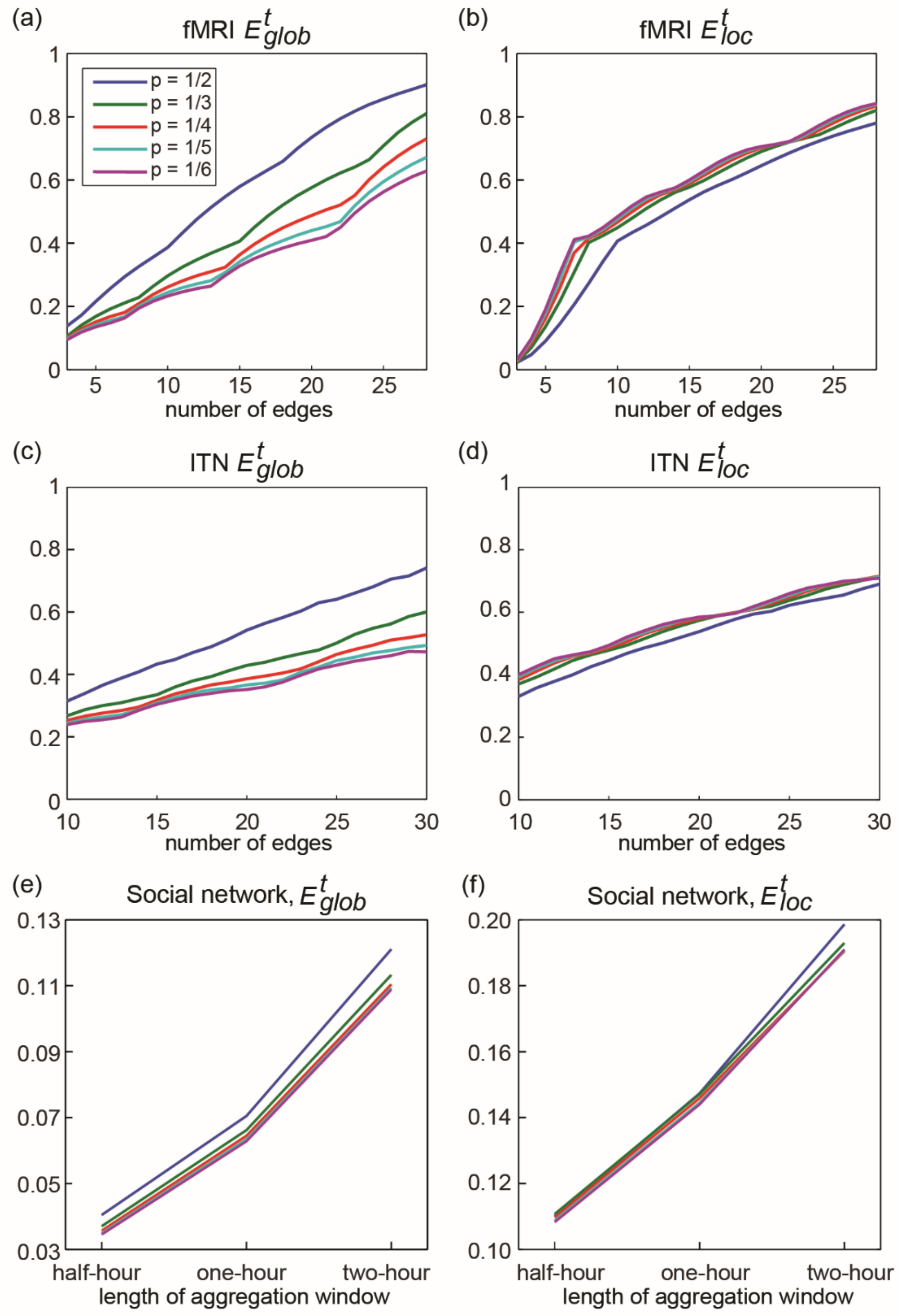

Figure S1. Temporal efficiency values of the temporal regular networks with different values of *p* in the network layer probability model, obtained using the fMRI functional connectivity networks (session 1) ((a) and (b)), the international trade networks ((c) and (d)), and the proximity networks ((e) and (f))

2) Network generation algorithm

Before the network generation process, the indices (coordinates) of all the contact positions are derived and grouped by layers, and the cumulative distribution function (CDF) of the layer probability distribution is

obtained. Afterwards, the temporal regular network is generated in two steps: (a) the number of contacts to be filled in each layer is derived by repeatedly sampling from the layer probability distribution (through inverse sampling using the CDF) until the desired total number of contacts (equal to that of the original network) are obtained; (b) for each layer, the corresponding number of contacts (derived from step (a)) are randomly distributed among all the positions belonging to the particular layer.

The pseudocode of temporal regular network and the layer generation algorithm are shown below.

Algorithm: Temporal regular network generation

Inputs: 1) Temporal network ***G*** ($N \times N \times T$), each network entry represents the presence (1) or absence (0) of a contact

2) Cumulative distribution function (CDF) of the layer probability distribution: ***cdf_layers*** ($N - 1 \times 1$)

3) Indices (coordinates) of all the contact positions in different layers: ***all_ind*** ($N - 1 \times 1$); each entry of ***all_ind*** is a matrix ($3 \times L$), where L is the maximum number of contacts in the current layer, and each column of the ($3 \times L$) matrix represents the 3-dimensional coordinate of one contact position

Outputs: Temporal regular network, $\boldsymbol{G_{reg}}$ ($N \times N \times T$)

Begin

Obtain the total number of contacts in ***G***: C;

% Step 1: generate the number of contacts to be filled in each layer

Calculate the maximum number of contacts in each layer: *layer_max* ($N - 1 \times 1$);

Initialize the counter of the number of filled contacts: $n = 0$;

Initialize an empty array to store the number of filled contacts in each layer: *layer_contact* ($N - 1 \times 1$);

While $n < C$

    Sample a layer number, $l \in 1:(N-1)$, from the distribution (***cdf_layers***) through inverse sampling;

    If *layer_contact*[*l*] < *layer_max*[*l*]

        layer_contact[*l*] = *layer_contact*[*l*] + 1;

        $n = n + 1$;

% Step 2: randomly fill the corresponding number of contacts in each layer

Initialize an empty matrix to store the new temporal regular network: G_{reg} ($N \times N \times T$);

For $l = 1:N - 1$

    Find the number of contacts to be filled in layer l: Nl = *layer_contact*[*l*];

    If $Nl > 0$

        Generate a randomly permuted integer sequence from 1 to *layer_max*[*l*]: r;

        Extract the first Nl elements of r: $s = r[1:Nl]$; *% r now represents the contacts to be filled in layer l*

        Find the Indices (coordinates) of corresponding contacts represented by r: *ind_l* = *all_ind*[*l*][1:3, *s*];

        Fill the contacts indexed by *ind_l* in G_{reg}: G_{reg}[*ind_l*] = 1;

Return G_{reg};

Algorithm: Layer generation for temporal regular network

Inputs: The number of nodes (*N*) and time steps (*T*) in the temporal network ***G***

Outputs: Coordinates of all the contact positions in ***G*** grouped by layers, ***all_layers*** (*N* - 1 × 1)

Begin

Derive the two functions representing the positions of the two space diagonals in ***G***: *diag_1* (*T* × 1), *diag_2* (*T* × 1):

diag_1(*t*) = *N* – floor[*N* + (-*N*) * (-*t*) / (-*T*)] – 1,

diag_2(*t*) = mod(*diag_1* + round(*N* / 2), *N*);

Initialize an empty cell to store the coordinates of different layers: *all_layers* (*N* - 1 × 1);

For *l* = 2 to *N*

Initialize an empty matrix to store all the coordinates belonging to the current layer: *layer* = [];

For *t* = 1 to *T*

Get the positions of the two diagonals at the current time step (*t*): *p1* = diag_*1*(t), *p2* = diag_*2*(t);

Initialize two empty vectors to store the row and column positions of the contacts: *r* = [], *c* = [];

For *ll* = 1 to (*l* - 1) *% (l - 1) is the equivalent "width" of layer (l - 1) in each static graph*

Locate the positions in the static graph at time *t* that belong to layer (*l* – 1), based on *p1* and *p2*;

Append the derived row index and column index to *r* and *c* respectively;

Remove the positions indexed by *r* and *c* that are occupied by previous layers;

Remove duplicate positions indexed by *r* and *c*, i.e., if (*r*[*i*], *c*[*i*]) == (*c*[*j*], *r*[*j*]), remove *r*[*i*] and *c*[*i*];

Concatenate *r* and *c* to *layer*;

Concatenate *layer* to *all_layers*;

Return *all_layers*;

III. Spatio-temporal view of all types of temporal networks (brain functional connectivity network)

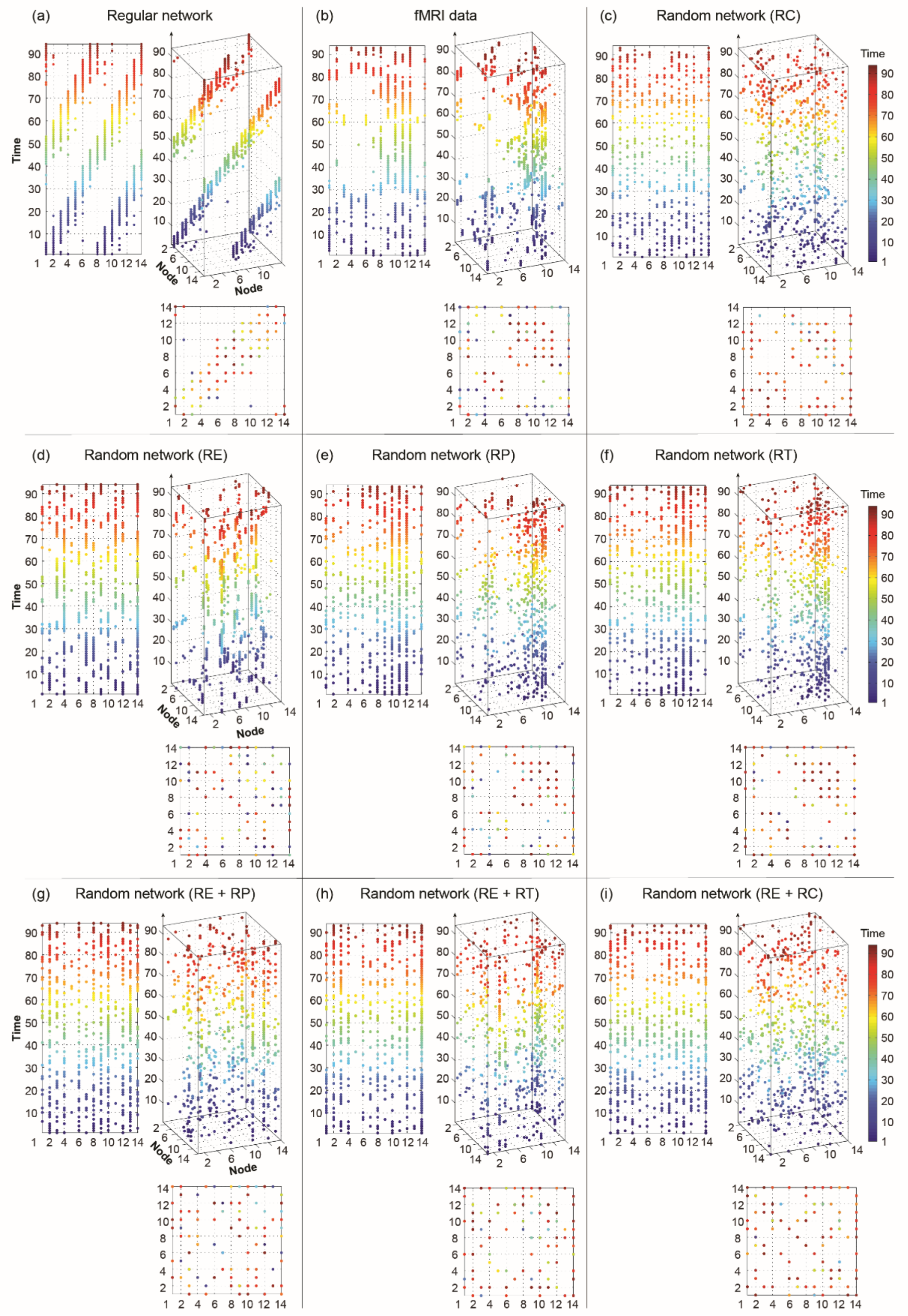

Figure S2. Spatio-temporal views of (b) temporal functional connectivity network, (a) the corresponding temporal regular network, and (c) – (i) the corresponding temporal random networks

IV. Construction of temporal brain functional connectivity networks

For the brain network analysis, we used the resting-state functional magnetic resonance imaging (fMRI) data recorded in a longitudinal study of the effect of mindfulness training in older adults with mild cognitive impairment (MCI). The study sample consisted of 17 subjects (age = 72.6 ± 5.6 [mean ± std] years, range: 64 – 86 years, male/female = 6/11) meeting the operational criteria of MCI[5], recruited from local communities. This study was approved by the Institution Review Board of National University of Singapore and was carried out in compliance with the Declaration of Helsinki. Each participant underwent two sessions of fMRI scanning, with an inter-session interval of approximately three months. During the three-month period, the participants were requested to perform mindfulness meditation training and awareness of internal and external experiences[6] for 40 minutes every day. The participants who failed to perform the continuous practice were excluded from the study. Subjects were scanned at rest on a 3-T Siemens Tim Trio scanner (Erlangen, Germany) at the Clinical Imaging Research Centre, Singapore. Subjects were instructed to keep still and remain as motionless as possible before the scanning. One high-resolution T1-weighted MRI volume images (repetition time [TR] = 2300 ms; echo time [TE] = 1.9 ms; matrix size = 256 × 256; voxel resolution = 1 × 1 × 1 mm^3) and one series of resting-state EPI images (310 volumes) were obtained axially (TR = 2300 ms; TE = 25 ms; flip angle = 90°; slice number = 48; thickness = 3 mm; field of view [FOV] = 192 × 192 mm^2; resolution = 64 × 64; voxel resolution = 3 × 3 × 3 mm^3).

Functional imaging data preprocessing was then performed using the SPM12 package (http://www.fil.ion.ucl.ac.uk/spm/software/spm12/), resting-state fMRI data analysis toolkit[7,8], which included the removal of the first 10 volumes, slice timing, head motion correction, nuisance signals regression, spatial normalization, and temporal band pass filtering[9]. We then employed a previously validated, automatically labelled template[10] to parcellate the brain into 90 regions of interest (ROIs), among which 14 ROIs representing the default mode network (DMN) were further selected in this work[11]. The representative time series of each ROI were obtained through averaging the time series of each voxel within the region. Next, temporal networks were established by applying an overlapping sliding window on the time series[12], calculating the Pearson's correlations between all pairs of nodes in each window, and applying different sparsity values (preserving the top n contacts in each static graph, n = 3, 4, ..., 28) to convert the matrices in each window into binary graphs. Here the window length was selected as 41.4s (18 TRs) to preserve the dynamics of the blood oxygen level dependent (BOLD) signals[13], with an incremental step of 6.9 s (3 TRs). As such, one temporal network (14 × 14 × 94) was obtained per subject at every sparsity value. For each of the temporal network, the corresponding temporal regular network and temporal random networks were generated, and E^t_{glob} and E^t_{loc} were calculated. Subsequently, the two efficiency measures in both sessions were integrated over the entire sparsity range to examine the presence of significant between-session differences.

V. Construction of temporal international trade networks

Using the United Nations commodity trade database (UN COMTRADE)[14], we built a temporal international trade network (ITN) consisting of 17 countries (including Argentina, Australia, Brazil, Canada, China, France, Germany, Indonesia, Italy, India, Japan, Rep. of Korea, Mexico, Saudi Arabia, Turkey, the United Kingdom and the United States) among the G20 countries whose trading data were consistently available during the span of 21 years (from 1994 to 2014). The network construction method is similar to that adopted in ref. 15. The trade flows were commodity-aggregated and the trade values (in US dollar) were used as the weights of the contacts in the network. The contacts were considered undirected (this assumption does not cause loss of significant topological information[16]). Here, in order to prevent repeated reports of the same trades, only the exports of each country to the other 16 countries were used. As a result, a single undirected weighted temporal network (17 × 17 × 21) was constructed. Then a series of sparsity values (preserving the top n contacts in each static network, n = 10, 11, ... 30) were applied to obtained different binary temporal networks (the assumption of binary contacts induces minimal loss of structural information[15]). For each of the resultant temporal network, the corresponding temporal regular and random networks were obtained and E^t_{glob} and E^t_{loc} were calculated.

VI. Integrated temporal efficiency measures

Table S1. Integrated (over the entire sparsity range) temporal efficiency measures of the brain networks, and the corresponding temporal random and regular networks

	Temporal network	Random networks							Regular network
		RE	*RP*	*RT*	*RC*	*RE + RP*	*RE + RT*	*RE + RC*	
E^t_{glob}, session 1	0.1501	0.2021	0.1978	0.1966	0.2110	0.2100	0.2089	0.2113	0.1059
E^t_{loc}, session 1	0.1173	0.0582	0.0863	0.0867	0.0755	0.0682	0.0686	0.0751	0.1511
E^t_{glob}, session 2	0.1505	0.2016	0.1985	0.1972	0.2106	0.2095	0.2085	0.2112	0.1059
E^t_{loc}, session 2	0.1210	0.0576	0.0883	0.0888	0.0752	0.0673	0.0678	0.0746	0.1511

Table S2. Integrated (over the entire sparsity range) temporal efficiency measures of the international trade networks (ITN), and the corresponding temporal random and regular networks

	Temporal network	Random networks							Regular network
		RE	RP	RT	RC	RE + RP	RE + RT	RE + RC	
E^t_{glob}	0.0890	0.1015	0.0933	0.0931	0.1055	0.1032	0.1031	0.1058	0.0624
E^t_{loc}	0.0721	0.0570	0.0696	0.0697	0.0641	0.0596	0.0597	0.0615	0.0891

VII. Construction of temporal social networks

The dataset used in the social network analysis is the Hypertext 2009 dynamic contact network data from the SocioPatterns project (http://www.sociopatterns.org/), which was recorded among the conference participants at the ACM Hypertext 2009 conference in Turin, Italy, from June 29 to July 1, 2009[17].

The data collection equipment is active Radio-Frequency Identification (RFID) devices embedded inside the conference badges, which is able to record face-to-face proximity data of the conference participants wearing the badges. When two persons stand closely facing each other (approximately 1.0 – 1.5 m away), ultra-low power radio packets are exchanged between the two devices carried by them, because the human body can act as RF shield at the carrier frequency used for communication. In this way, interpersonal physical proximity (contact) can be detected with a high probability (over 99%) at a fine temporal resolution (20 s). After the establishment of a particular contact, the durations of the contact can be approximated by observing the number subsequent 20 s-intervals in which at least one signal exchange is detected. More details on the data collection facility and process are described in (http://www.sociopatterns.org/) and ref. 17-20. 113 conference attendees (around 75% of all the participants) took part in the data collection, corresponding to 113 nodes in the temporal networks. The data recording spanned approximately 59 hours.

Due to the sparse nature of the human proximity network under study (average sparsity over all time steps < 0.01%) and consideration of the computational cost, we applied aggregation time windows (referred to as snapshot rate in ref. 21) on the data to obtain aggregated temporal networks. The contacts were aggregated in such a way that a (binary) link exists between two persons in the aggregated network if at least one contact occurred within the current time window. Ideally, the time scale of network evolution, which in this case is determined by the aggregation time window, should be of the same order with, or shorter than, the characteristic time scale of the dynamics unfolding on the network[2]. On the other hand, given the absence of characteristic time scale in the temporal proximity data under study[17,18], different time windows could reveal complementary aspects of the network dynamics[22]. In order to investigate the impact of different aggregation windows on the network characteristics, we applied three different time windows: half-hour, one-hour and two-hour windows. The windows with no contacts were removed, and very brief contacts (with only one contact within an aggregation period) were eliminated from further network analysis[2], resulting in 72 time steps in half-hour window, 38 time steps in one-hour window, and 20 time-steps in two-hour window conditions.

For each of the three resultant temporal networks, temporal random and regular networks were generated and temporal efficiency values were calculated for all the networks, following which the presence of temporal small-world architecture was explored. Subsequently, we investigated a particular network characteristic in the temporal proximity networks: the degree of similarity between the snapshot networks at adjacent time steps, which we refer to as temporal persistence in this work. Specifically, the impacts of the temporally persistent network structure on the temporal efficiency measures were quantitatively inspected through comparisons with the temporal random networks under *RP* randomization, which mitigates the temporal persistence in the networks while preserving most of the network structures. Moreover, the effects of the aggregation time window on the temporal efficiencies were explored, and interpreted through examining two additional network characteristics:

average degree and adjacency correlation. The average degree measures the mean degree of all the nodes averaged over the network lifetime, while the adjacency correlation measures the similarity between the network snapshots at adjacent time steps[21].

VIII. Results and discussion of temporal social networks

The temporal efficiencies of the proximity networks with different aggregation windows, together with those of the corresponding temporal random and regular networks, are shown in Fig. S3. As shown in the figure, comparisons between the efficiency measures of the proximity networks and those of the temporal random (with *RE* + *RC* randomizations) and regular networks revealed the presence of optimal temporal small-world structure in all three networks. Therefore, both the global interactions amongst all the conference attendees as well as the local interpersonal communications within small neighborhoods are organized to be efficient.

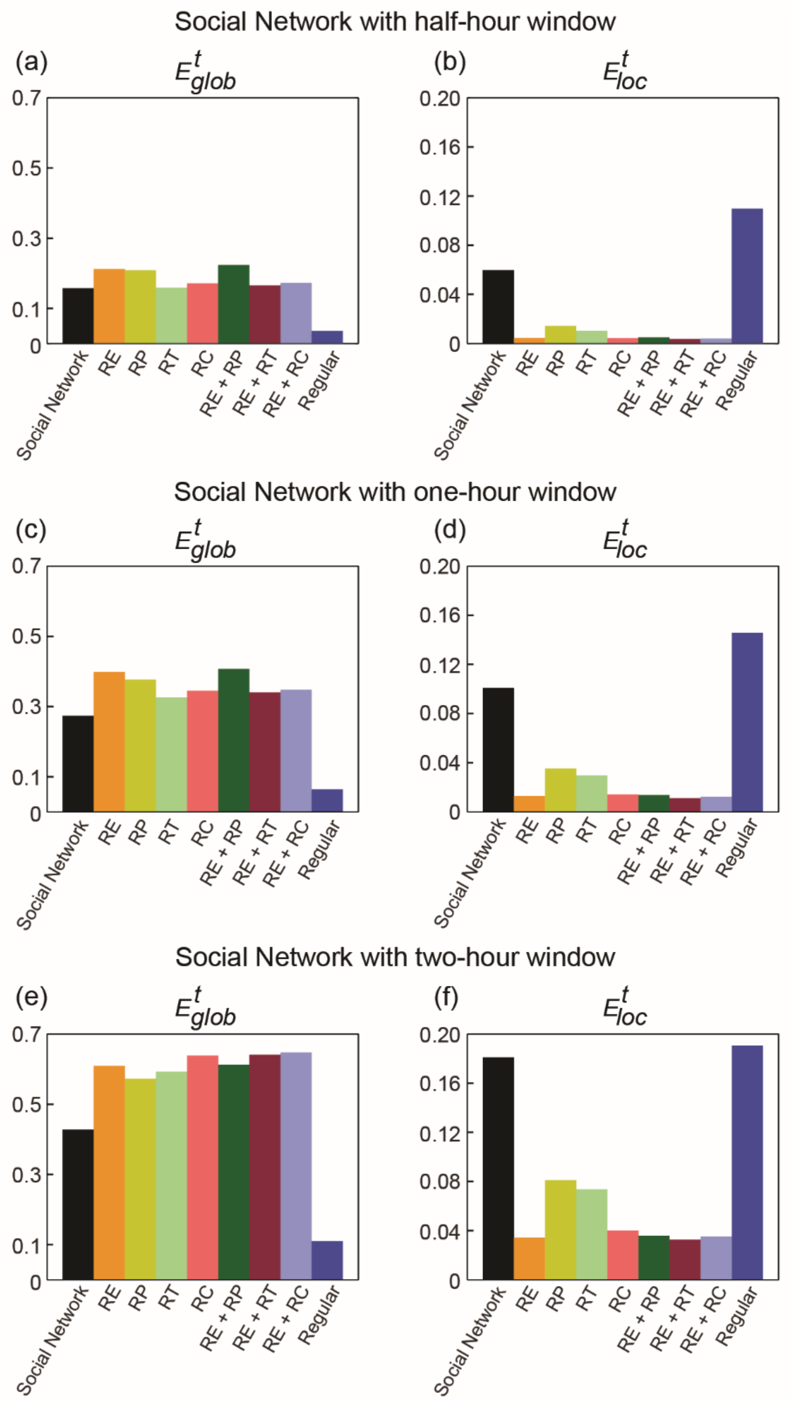

Figure S3. Overall temporal global and local efficiency of the temporal proximity networks constructed with half-hour ((a) and (b)), one-hour ((c) and (d)) and two-hour aggregation window ((e) and (f)), and those of the corresponding temporal regular network and the corresponding temporal random networks.

We then investigated the impact of the temporally persistent structure in social networks on the temporal efficiency measures. The adjacency correlations of the proximity networks and the corresponding random networks with *RP* randomization are shown in Table S3. It can be observed in the table that, compared with the original networks, the adjacency correlations of the *RP*-randomized networks (averaged over 100 iterations) were

consistently reduced for all time windows, suggesting that *RP* randomization decreases the temporal persistence in the proximity networks. Meanwhile, as shown in Fig. S3, for all three temporal proximity networks, the temporal reference networks under *RP* randomization exhibited significantly improved E^t_{glob} and declined E^t_{loc}. These two observations might indicate that the temporally persistent network structure (specifically, relatively high degree of similarity between adjacent snapshot networks), which is alleviated in the *RP*-randomized networks, may deflate E^t_{glob} and inflate E^t_{loc}. In temporally persistent networks, the topologies of adjacent snapshot graphs are fairly similar, therefore, only a small number of new time-respecting paths are created by traveling an additional time step; as a result, it takes relatively more time steps for a node to reach every other node in the network through time-respecting paths, thus reducing the overall temporal global efficiency of the networks. On the other hand, the strong similarity between the topologies of adjacent snapshot graphs causes the densely connected local communities to be the stable over time, facilitating effective communication among neighboring nodes and therefore boosting the overall temporal local efficiency.

Table S3. Adjacency correlations of the human proximity networks and the corresponding RP-randomized networks (averaged over 100 iterations) with different aggregation time windows

	Half-hour network	One-hour network	Two-hour network
Adjacency correlation, proximity network	0.9074	0.8397	0.7446
Adjacency correlation, *RP*-randomized network	0.8571	0.7693	0.6409

In the modeling process of temporal networks, time window can have substantial impacts on the network characteristics and therefore caution is needed in choosing the appropriate time window[2,21]. Table S4 shows the E^t_{glob} and E^t_{loc} of the three temporal proximity networks with different aggregation windows. As shown in the table, both E^t_{glob} and E^t_{loc} were increased with lengthening aggregation window. In the meantime, as shown in Table S5, longer aggregation window increases the average degree of the networks. Additionally, as shown in Table S3, prolonged time window leads to decreasing adjacency correlation (the same pattern in proximity network is discovered in ref. 21), which will contribute to the inflation of E^t_{glob} and deflation of E^t_{loc} according to the previous analysis. Taking into account these findings, we conjecture that the increment in E^t_{glob} and E^t_{loc} resulting from the prolonged aggregation window might be mostly explained by the increasing average degree, i.e., the increasing density of connections in the network. This interpretation can be further corroborated by the observations from the brain network (Fig. 5) and international trade network (Fig. 7), in which both E^t_{glob} and E^t_{loc} were monotonically increased with increasing number of preserved contacts in the networks.

Table S4 Overall temporal global and local efficiency values of the human proximity networks with different aggregation time windows

	Half-hour network	One-hour network	Two-hour network
E^t_{glob}	0.1571	0.2735	0.4274
E^t_{loc}	0.0596	0.1007	0.1809

Table S5. Average degree of the human proximity networks with different aggregation time windows

	Half-hour network	One-hour network	Two-hour network
Average degree	0.5650	0.9718	1.7063

Taking together all the observations above, we conclude that: *1)* the physical proximity networks under study possess temporal small-world architecture; *2)* the temporally persistent structure in social networks might deflates the overall temporal global efficiency, while inflating the overall temporal local efficiency; *3)* longer aggregation window in the proximity network analysis increases the density of connections, which might result in larger temporal network efficiencies both globally and locally.

IX. Static vs. temporal small-world networks

We also studied the correspondence between static small-worldness (as defined in ref. 23) and temporal small-worldness (as defined in this work), based on the temporal randomization techniques introduced in this work. Specifically, we explored whether static small-worldness of the aggregated static network has any implication on the temporal small-worldness of the underlying temporal network, i.e., *1)* whether the underlying dynamic network is necessarily temporally small-world if the aggregated network is a static small-world network, and *2)* whether it is possible for the temporal network to possess temporal small-worldness if static small-worldness is absent in the aggregated network.

Firstly, we examined whether static small-worldness would necessarily indicate that the underlying dynamic network is also temporally small-world. In static networks, the efficiency of information flow depends only on the network topology, making it the single factor determining whether the network is small-world. In contrast, in temporal networks, an additional dimension (time) is incorporated, and both the topological and temporal network structures combine to influence the efficiency of information exchange on the networks, and consequently to determine the temporal small-worldness of the networks. Therefore, for an aggregated static network with optimal small-world topology, the information communication efficiency of the underlying temporal network is highly dependent on the temporal configurations of the network (e.g., the order of occurrence of the contact events), making the presence of temporal small-worldness not guaranteed. As a verification, we explored the temporal and static small-worldness of the proximity network with one-hour aggregation window. Firstly, we aggregated the proximity network over the entire network lifetime to obtain the static proximity network; subsequently, following similar procedures to those in ref. 24, we evaluated the static small-worldness through comparing the global and local efficiency[25] of the static proximity network with those of the corresponding random and regular networks (both averaged over 100 iterations) (Table S6). As shown in Table S6, the static proximity network exhibited both large global efficiency (compared with regular network) and large local efficiency (compared with random network). Moreover, the aggregated proximity network of the same dataset is also discovered to be statically small-world in ref. 17. Secondly, in terms of the underlying temporal networks, both the original temporal proximity network and the *RP*-randomized network have the same aggregated static network, because *RP* randomization preserves the aggregated topological structure of the original temporal network; however, as shown in Fig. 8, the proximity network is temporally small-world, while the *RP*-randomized network is temporally non-small-world. To conclude, given a small-world aggregated static network, the underlying temporal network can be either temporally small-world or temporally non-small-world.

Table S6 Global and local efficiency of aggregated static proximity network, and the corresponding random and regular networks (both averaged over 100 iterations)

	Aggregated proximity network	Random network	Regular network
Global efficiency	0.5632	0.5644	0.2513
Local efficiency	0.6416	0.6074	0.8277

Note: The global and local efficiency refer to the efficiency definitions in static graphs[25].

Next, we explored if it is possible for the underlying dynamic network to possess temporal small-worldness if the aggregated network is statically non-small-world. As described previously, temporal small-world architecture is facilitated by the combination of special topological and temporal structures in the network, both of which are key ingredients for the temporal small-worldness. Therefore, the removal of optimal topological structure from the aggregated network might eliminate of the possibility for the underlying dynamic network to be temporally small-world. In order to verify this conjecture, we employed the *RE* randomization technique, which destroys the topological structure of the temporal network while preserving the temporal structure. In the *RE* technique, the randomization procedures performed on the aggregated network are the same as those in ref. 4, which is used to produce static random network. As a result, if a temporal network is randomized with *RE*, the aggregated graph of the resultant network is guaranteed to be statically non-small-world; meanwhile, for all three types of real-world dynamic systems investigated in this study, all the *RE*-randomized networks are temporally non-small-world (Fig. 5, 7, 8 and Fig. S3). Therefore, we hypothesize that if an aggregated static network is non-small-world, the underlying temporal network is guaranteed to be temporally non-small-world.

To summarize, we hypothesize that static small-worldness of the aggregated network is a **necessary but not sufficient** condition for temporal small-worldness of the underlying temporal network.

References

1 Holme, P. Analyzing temporal networks in social media. *Proc. IEEE* **102**, 1922-1933, doi:10.1109/JPROC.2014.2361326 (2014).

2 Holme, P. Modern temporal network theory: a colloquium. *Eur. Phys. J. B* **88**, 1-30, doi:10.1140/epjb/e2015-60657-4 (2015).

3 Holme, P. & Saramäki, J. Temporal networks. *Phys. Rep.* **519**, 97-125, doi:10.1016/j.physrep.2012.03.001 (2012).

4 Maslov, S. & Sneppen, K. Specificity and stability in topology of protein networks. *Science* **296**, 910-913, doi:10.1126/science.1065103 (2002).

5 Petersen, R. C. Mild cognitive impairment as a diagnostic entity. *J. Int. Med.* **256**, 183-194, doi:10.1111/j.1365-2796.2004.01388.x (2004).

6 McBee, L. *Mindfulness-based elder care: A CAM model for frail elders and their caregivers*. (Springer Publishing Co, 2008).

7 Song, X. W. *et al.* REST: a toolkit for resting-state functional magnetic resonance imaging data processing. *PLoS. One.* **6**, e25031, doi:10.1371/journal.pone.0025031 (2011).

8 Chao-Gan, Y. & Yu-Feng, Z. DPARSF: A MATLAB Toolbox for "Pipeline" Data Analysis of Resting-State fMRI. *Front. Syst. Neurosci.* **4**, 13, doi:10.3389/fnsys.2010.00013 (2010).

9 Sun, Y. *et al.* Disrupted functional brain connectivity and its association to structural connectivity in amnestic mild cognitive impairment and Alzheimer's disease. *PLoS. One.* **9**, e96505, doi:10.1371/journal.pone.0096505 (2014).

10 Tzourio-Mazoyer, N. *et al.* Automated anatomical labeling of activations in SPM using a macroscopic anatomical parcellation of the MNI MRI single-subject brain. *Neuroimage* **15**, 273-289, doi:10.1006/nimg.2001.0978 (2002).

11 Liu, F. *et al.* Multivariate classification of social anxiety disorder using whole brain functional connectivity. *Brain Struct. Funct.* **220**, 101-115, doi:10.1007/s00429-013-0641-4 (2015).

12 Calhoun, V., Yaesoubi, M., Rashid, B. & Miller, R. in *Global Conference on Signal and Information Processing (GlobalSIP), 2013 IEEE.* 831-834 (IEEE).

13 Zalesky, A. & Breakspear, M. Towards a statistical test for functional connectivity dynamics. *Neuroimage* **114**, 466-470, doi:10.1016/j.neuroimage.2015.03.047 (2015).

14 Comtrade, U. United Nations commodity trade statistics database. *URL: http://comtrade. un. org* (2010).

15 Squartini, T., Fagiolo, G. & Garlaschelli, D. Randomizing world trade. I. A binary network analysis. *Phys. Rev. E* **84**, doi:10.1103/PhysRevE.84.046117 (2011).

16 Serrano, M. A. & Boguna, M. Topology of the world trade web. *Phys. Rev. E.* **68**, 015101, doi:10.1103/PhysRevE.68.015101 (2003).

17 Isella, L. *et al.* What's in a crowd? Analysis of face-to-face behavioral networks. *J. Theor. Biol* **271**, 166-180 (2011).

18 Cattuto, C. *et al.* Dynamics of person-to-person interactions from distributed RFID sensor networks. *PLoS. One.* **5**, e11596, doi:10.1371/journal.pone.0011596 (2010).

19 Alani, H. *et al.* in *International Semantic Web Conference.* 698-714 (Springer).

20 Van den Broeck, W. *et al.* in *Pervasive Computing and Communications Workshops (PERCOM Workshops), 2010 8th IEEE International Conference on.* 226-231 (IEEE).

21 Clauset, A. & Eagle, N. Persistence and periodicity in a dynamic proximity network. *arXiv preprint arXiv:1211.7343* (2012).

22 Holme, P. & Saramäki, J. (Springer Berlin Heidelberg, 2013).

23 Watts, D. J. & Strogatz, S. H. Collective dynamics of 'small-world' networks. *Nature* **393**, 440-442, doi:10.1038/30918 (1998).

24 Achard, S. & Bullmore, E. Efficiency and cost of economical brain functional networks. *PLoS. Comput. Biol.* **3**, e17, doi:10.1371/journal.pcbi.0030017 (2007).

25 Latora, V. & Marchiori, M. Efficient behavior of small-world networks. *Phys. Rev. Lett.* **87**, 198701, doi:10.1103/PhysRevLett.87.198701 (2001).
